# Round the Hospitals

**Published:** 1916-02-26

**Authors:** 


### Round the Hospitals.

The war has brought many changes and more vicissitudes for those members of the nursing profession who have been most anxious to become associated with war work. We have received a letter from an old St. Thomas's Hospital probationer, who remained ten years at St. Thomas's, of which she was home sister for seven years, and afterwards was matron for six years of two hospitals successively, one in Lancashire and one in London, being subsequently in charge for seven years of important nursing homes, making twenty-three years' service in all. Anxious to get to the Front, she accepted employment as staff nurse in Queen Alexandra's Imperial Military Nursing Service Reserve. She is now working at the Front, and has found time to write a letter expressing her approval of the proposal to establish a College of Nursing. This case affords evidence of the want of business management in the affairs of the War Office—*i.e.*, in those responsible there for the administration and working of Queen Alexandra's Imperial Military Nursing Service and its Reserve, and the utilisation of its members. From the point of view of efficient administration what justification can there be for sending an experienced matron, who for patriotic reasons joined the Reserve, to the Front as a staff nurse, when efficient matrons are none too plentiful?

We gave a striking instance last week of faulty responsibility in the absence of the Matron-in-Chief from St. Paul's Cathedral when the Queen attended to unveil the Florence Nightingale Memorial. It is remarkable, seeing how many able women there are available, that those who are responsible for the direction of nursing affairs for the Army do not exert themselves continuously to secure that none but the best service shall find any place whatever in the higher posts. It is currently reported that Miss Becher was to have retired before the war, and only remained for the period of the war. Surely, during strenuous times like the present, it is obvious that the direction and control of military nursing should be placed in the hands of the most competent, active, and gracious personage the Service can provide.

Miss Isabella M. Ewins, sister-in-charge of the Ida and Arthington Hospital (the semi-convalescent branch of the Leeds General Infirmary, Cookridge), who was trained at the Birmingham Hospital for Women and the Leeds General Infirmary, has been appointed, out of thirty-eight applicants, matron of the Barnes Convalescent Hospital, Cheadle, Cheshire, which is under the management of the Royal Infirmary, Manchester. Miss Ewins did good service at the Leeds General Infirmary as sister of the male ophthalmic ward, sister of the gynæcological ward, and later as night superintendent and home sister. We congratulate the Manchester Royal Infirmary upon their choice, for we found the semi-convalescent branch at Cookridge most excellently managed and disciplined when we inspected it, and have always regarded it as one of the most useful types of semi-convalescent hospitals. Every great voluntary hospital throughout the country should have its semi-convalescent hospital.

An esteemed correspondent explains that the matron has not sent items of interest regularly, as she is never sure how far she is justified in taking for granted that they will be welcome. We hope every one of our readers, both matrons and sisters, will accept this intimation—that every item of interest is always welcome and always wanted.

---

The Hospital has always held that in cases where a mild cigarette tends to help a woman in her work it is to be encouraged. A correspondent on page 463 of The Hospital of last week raises a totally different point, on which we invite correspondence. The problem she submits is whether matrons should allow their sisters and nurses to smoke. Circumstances alter cases, and is it conceivable that any matron would venture to sanction smoking by sisters or nurses in hospital corridors or wards? We understand, thiugh we cannot credit the statement, that there is one matron at least who frequently smokes cigarettes when making her rounds. Not long ago cigarette-smoking in the Indian Nursing Service was reported to have reached the point of scandal in one or more centres. Many V.A.D. workers are reported to smoke, if not to excess, at least beyond the limits of discretion in the case of young women working in an institution. Where the standard of conduct is of the highest, we have never before, in forty-five years, heard a suggestion even that cigarette-smoking is desired, let alone habitually practised. We invite correspondence that the actual position of matters as they are to-day may be made apparent and a standard set up.

---

A matron of high standing sends us the following thoughts: "In concluding the leading article in your issue of January 22 on 'Honours and the Nursing Profession,' you state that 'it is impossible to include in the present Honours List every worker whose services merit distinction.' The truth of this statement is obvious, and along the present lines the difficulties of appropriate selection are necessarily very great. It is scarcely possible in the performance of their duties for individual nurses to distinguish themselves in the same way as soldiers, whose conspicuous bravery may merit such honours as the Victoria Cross or the D.S.O. The services rendered by the profession must be recognised in some way in which the honours bestowed are representative. Thus the recognition of the matron of a hospital, or centre of hospitals, confers honour on the whole unit for which she stands, and all the members of her staff share in her distinction. The selection of one or more nurses from the staff of a hospital, however, might almost be thought to constitute a reflection on the work of the remainder, and from a wide knowledge of nurses I know that decorations thus bestowed on the rank and file, far from gratifying the other nurses, are regarded as invidious distinctions, and cause discontent, and even jealousy, amongst those who have been passed over. Speaking as one whom it did not affect, I should like to say that I altogether approved the recognition of the principal matrons of the Territorial Hospitals, as it was an acknowledgment of the work done in this country, and further, I think that the matrons of the civil hospitals having military wards should not be overlooked." What is the general view of the position as stated?

---

Many of the great voluntary hospitals in the provinces have a splendid war record. Thus the Royal Infirmary, Liverpool, out of 142 nurses, inclusive of the private staff, has sent forty sisters and nurses to the seat of war, including the Western Front, Malta, Egypt, and hospital ships. Twenty-one of these nurses are engaged at present in the hospitals at Malta. No fewer than fourteen out of eighteen sisters have been granted leave of absence for war service since August 1914. Miss Margaret Cummins has organised a very practical scheme for training V.A.D. nurses in the discharge of their duties by arranging classes for three months' instruction, and 160 V.A.D.s have already passed out into active service. Another practical and useful innovation at Liverpool has been a plan whereby Girl Guides have been trained in the kitchen and put to work in the Home so as to qualify them for a certificate of efficiency. It is interesting to note that the new tropical ward at the Royal Infirmary has been lent to the Admiralty for the accommodation of sailors, whilst the soldier patients are now accommodated in 330 beds specially set apart for their use.

---

Since the war began there has been much talk of "dug-outs," at times with much reason no doubt, and of people too old for war work. A prolonged period of peace, with increasing comfort and even luxury in the majority of homes, in a nation like ours tends to promote dug-out or wait-and-see principles in members of both sexes. Ability to take part in the war does not depend so much upon age as upon character. The spirit to do and to take one's part in defence of one's country, if present, becomes in practice superior even to habit, and may even change a dug-out into a useful worker within the natural limitations of the individual. It is interesting in this connection in view of the tendency constantly to depreciate candidates over forty years of age for nursing positions and service, to remember that Clara Barton, the American "angel of the battlefield," was forty years of age when she began her life-work for the alleviation of suffering. Unless dug-out incompetence is a result of long routine service under Army conditions, its victim may have spirit enough left in war-time to become a man and a patriot. Amongst women, our Nursing Service during the last year and a half has revealed how few of them are dug-outs, and, where they exist, how true is our reference to the Army dug-out type.

---

Will each matron and sister interested cooperate with the Editor of The Hospital and do her part to make this Department of Nursing of practical use and interest each week?

